# DCRELM: dual correlation reduction network-based extreme learning machine for single-cell RNA-seq data clustering

**DOI:** 10.1038/s41598-024-64217-y

**Published:** 2024-06-12

**Authors:** Qingyun Gao, Qing Ai

**Affiliations:** School of Computer Science and Software Engineering, University of Science and Technology Liaoning, Anshan, 114051 China

**Keywords:** ScRNA-seq data, Deep clustering, Extreme learning machine, Dual correlation information reduction, Feature fusion, Machine learning, Data mining, Computational models

## Abstract

Single-cell ribonucleic acid sequencing (scRNA-seq) is a high-throughput genomic technique that is utilized to investigate single-cell transcriptomes. Cluster analysis can effectively reveal the heterogeneity and diversity of cells in scRNA-seq data, but existing clustering algorithms struggle with the inherent high dimensionality, noise, and sparsity of scRNA-seq data. To overcome these limitations, we propose a clustering algorithm: the Dual Correlation Reduction network-based Extreme Learning Machine (DCRELM). First, DCRELM obtains the low-dimensional and dense result features of scRNA-seq data in an extreme learning machine (ELM) random mapping space. Second, the ELM graph distortion module is employed to obtain a dual view of the resulting features, effectively enhancing their robustness. Third, the autoencoder fusion module is employed to learn the attributes and structural information of the resulting features, and merge these two types of information to generate consistent latent representations of these features. Fourth, the dual information reduction network is used to filter the redundant information and noise in the dual consistent latent representations. Last, a triplet self-supervised learning mechanism is utilized to further improve the clustering performance. Extensive experiments show that the DCRELM performs well in terms of clustering performance and robustness. The code is available at https://github.com/gaoqingyun-lucky/awesome-DCRELM.

## Introduction

scRNA-seq is a technique for sequencing and analysing the genome or transcriptome at the single-cell level. This approach reveals heterogeneity and diversity among cell populations and allows the analysis of gene sequences and expression within the transcriptome range^1–3^, which is crucial for investigating large-scale cell atlases^4^ and complex diseases^5–7^ and for characterizing cell types^8,9^.

scRNA-seq technology has been widely applied in various fields of biology and medicine^10–12^. Cell clustering is a crucial step in scRNA-seq data analysis. Clustering of cells groups similar cells into different cell clusters, which helps us identify different types, subtypes, and states of cells, facilitating a better understanding of the diversity and function of cells. Moreover, effective identification of cell types affects the downstream analysis of scRNA-seq data^1,2^. Therefore, many clustering algorithms, such as spectral clustering^13^, k-means^14,15^, Celltree^16^, and Gaussian mixture models (GMMs), are used to identify cell types^17^. Transcriptional bursting refers to the activation signal that promotes genes to transition from a silent state to an active state, rapidly initiating transcription, generating a large amount of mRNA within a short period, and then returning to a silent state^18^. In the early discovery of transcriptional bursting, it was widely regarded as noise^19^. The single-cell transcriptional bursting effect is caused by the randomness and noise of transcription. During the transcription process, gene expression undergoes random bursts of enhancement or suppression, resulting in transcriptional differences among cells. As a result, scRNA-seq data are sparser, leading to the majority of measurements being zero. The most prominent phenomenon is the dropout event, where low RNA capture rates yield false or close zero values of gene expression in some cells^20–22^. In addition, scRNA-seq data exhibit a high degree of variability at the gene expression level. To address this problem, a significant amount of noise arises due to biological and technical variations. Therefore, effective scRNA-seq data clustering algorithms are crucial.

In recent years, single-cell clustering algorithms have been proposed to address the challenges associated with scRNA-seq data. SIMLR^23^ utilizes a multikernel learning framework to capture the complex relationships among cells based on gene expression profiles. SC3^24,25^ addresses cell heterogeneity by integrating multiple clustering results to obtain a consensus clustering solution. CIDR^26^ is an ultrafast algorithm for clustering by inference and dimensionality reduction that uses implicit inference to interpolate zeros during distance computation to reduce the impact of dropout in scRNA-seq data. These methods yield good clustering performance but have high computation and storage costs and suffer from high complexity and limited scalability.

Due to the excellent performance of deep learning, numerous deep clustering techniques have been introduced by researchers for scRNA-seq data analysis. scDeepCluster^20^ can utilize the representation learning ability of deep autoencoders to capture complex patterns in scRNA-seq data. By combining deep learning and clustering, deep learning methods can handle high-dimensional data. ADCluster^27^ can simultaneously achieve anomaly detection and clustering analysis, aiming to identify outliers in the dataset and cluster normal samples. scCAEs^28^ is a scRNA-seq clustering algorithm that utilizes convolutional autoencoder embedding and soft K-means deep embedding and can learn latent clustered cell populations in space. scDCCA^29^ employs denoising autoencoders (DAEs) and a contrastive learning module to extract valuable features. scDASFK^30^ uses DAEs and self-attention mechanisms within a comparative learning framework to improve its robustness and extract additional critical features. DREAM^31^ combines a variational autoencoder and GMM to visually analyse scRNA-seq data while introducing a zero-inflated layer for dimensionality reduction. These algorithms based on autoencoders (AEs) focus on analysing the data and do not explicitly consider the relationships among cells or the intrinsic characteristics of the cells. Consequently, the algorithms cannot effectively learn features.

Most existing methods rely primarily on gene expression information during the representation learning process and do not explicitly share topological information among cells. To capture the complex relationships among cells and their intrinsic properties, several novel algorithms based on graph neural networks (GNNs), such as the scGNN^32^, scGAC^33^, GraphSCC^34^, and scDFC^35^ algorithms, have been proposed. The scGNN combines graph convolutional networks (GCNs)^36^ with single-cell clustering to capture complex relationships among cells. However, the constructed graphs may contain noise connecting different types of cells, thus leading to differences in cell types that may be confused and hence misleading the clustering results. scGAC overcomes these limitations by utilizing a graph attentional autoencoder to learn the latent representation of cells. GraphSCC solves the higher-order structural relationships among cells. scDFC uses attribute information and attention mechanism-based structural information to accurately construct cell-to-cell graphs to address complex biological situations. However, existing GNN-based clustering methods often suffer from representation collapse and tend to map nodes of different categories to similar representations during the cell-gene expression encoding process, making them ineffective at distinguishing different types of cells.

To overcome these issues, we propose a dual correlation reduction network-based extreme learning machine (DCRELM). First, the scRNA-seq data are mapped to low-dimensional and dense result features through the ELM random mapping space. The ELM graph distortion module is used for data augmentation in the feature space and structural space to improve the robustness of the model. Second, in the autoencoder fusion module, the AE and IGAE dynamic fusion mechanisms are used to obtain consistent latent representations, and the dual information correlation reduction module filters redundant information and noise. Last, a triplet self-supervised learning mechanism is employed to further improve the clustering performance.

## Materials and methods

### Datasets

We constructed comparative experiments on 12 real scRNA-seq datasets to verify the effectiveness of our DCRELM. The detailed information of these datasets is shown in Table 1, where #Cell is the number of cells, #Genes is the number of genes, #Cell types is the number of cell subtypes, and #References is the source of the dataset. We use datasets with small, medium, and large-scale samples, as well as datasets with significant features ranging from low to high dimensions.

**Figure 1:**
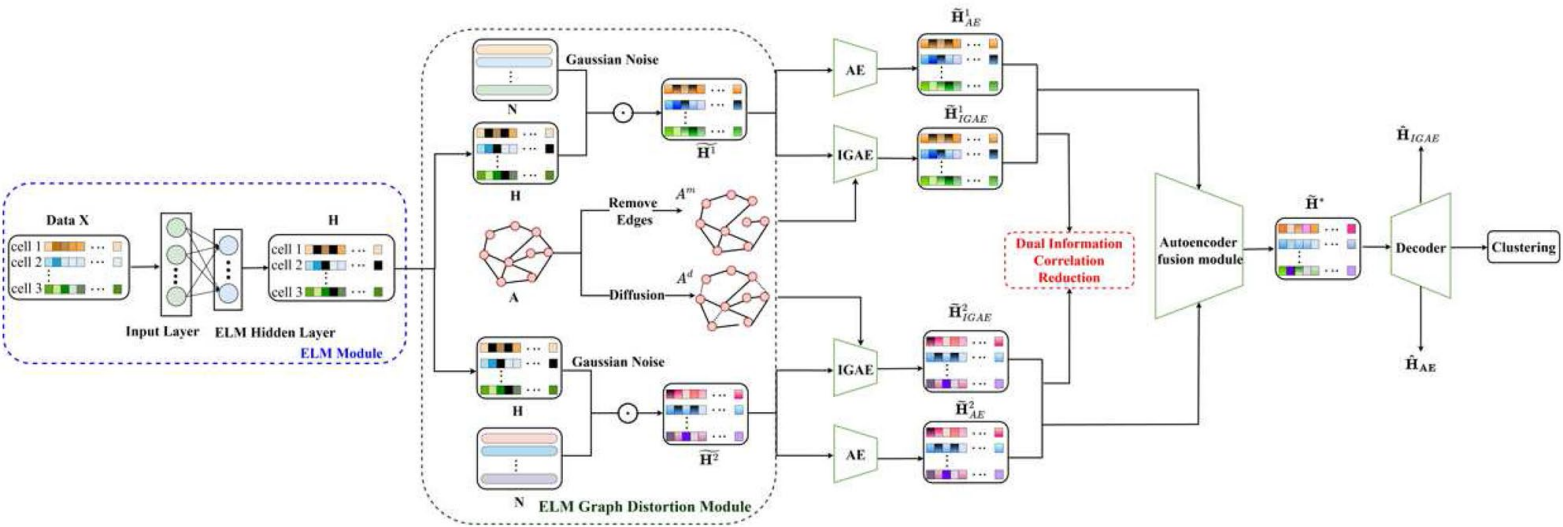
Framework diagram of the DCRELM. The cell-gene expression matrix *X* from the scRNA-seq data was selected as the input matrix. The ELM maps the original high-dimensional sparse *X* into a random mapping space to obtain a low-dimensional dense cell output matrix *H*. Using a siamese network framework, the attribute information of the cell output matrix *H* is enhanced to obtain ${\widetilde{H}}^1$ and ${\widetilde{H}}^2$, while the graph structure information of the cell adjacency matrix *A* is enhanced to obtain $A^m$ and $A^d$. Then, the fusion encoder in the autoencoder fusion module is employed to extract latent features $H^{\upsilon 1}$ and $H^{\upsilon 2}$ from ${\widetilde{H}}^1$ and ${\widetilde{H}}^2$, and the dual correlation information reduction network is utilized to remove noise and redundant feature information. High-quality cell-gene expression features are obtained by decoding the fusion module. The KL loss function of the triplet self-supervised strategy is minimized to improve clustering performance and effectively identify cell types.

**Figure 2:**
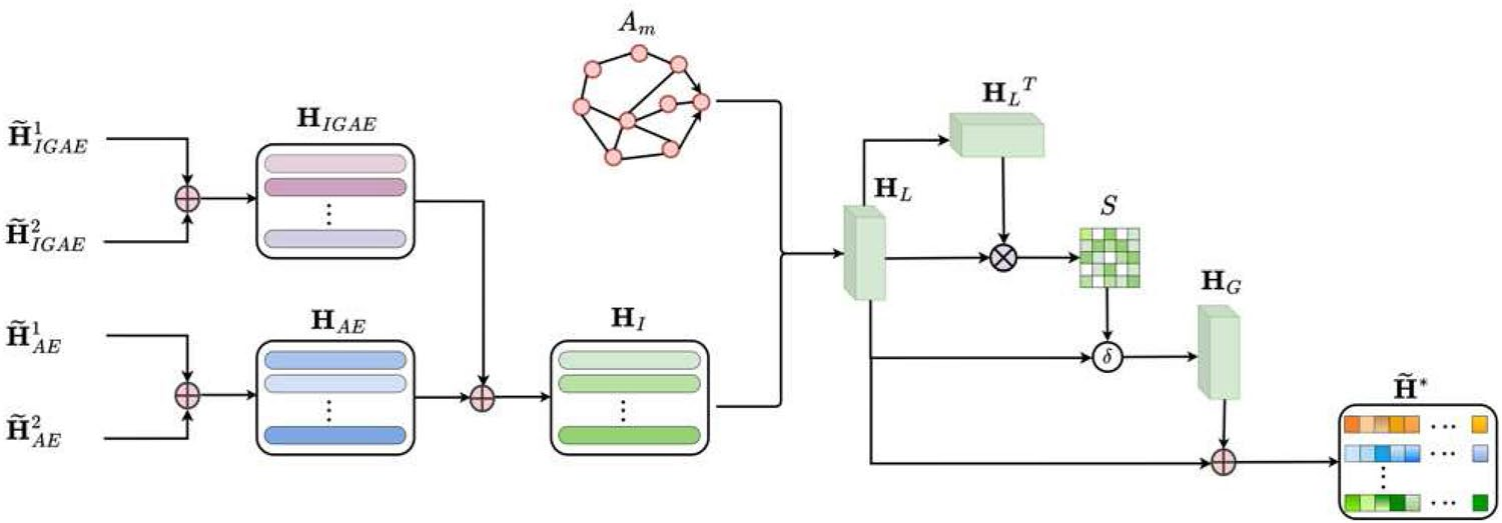
Flowchart of the autoencoder fusion module. The autoencoder fusion module obtains the attribute information ${\widetilde{H}}_{AE}^{1}$ and ${\widetilde{H}}_{AE}^{2}$ of cells and the graph structure information ${\widetilde{H}}_{IGAE}^{1}$ and ${\widetilde{H}}_{IGAE}^{2}$ among cells via AE and IGAE and fuses these two pieces of information to obtain more suitable feature representations ${\widetilde{H}}^*$ of cells.

**Table 1:** Characteristics of experimental datasets.

Dataset	#Cell	#Genes	#Cell types	Repositories	Accession numbers
Human	124	3840	8	Gene expression omnibus	GSE36552
Yeo	214	34608	4	Gene expression omnibus	GSE85908
Ning	460	19084	4	Gene expression omnibus	GSE64016
Lawor	638	26616	8	Gene expression omnibus	GSE86473
Kolodziejczy (Kolo)	704	2000	3	ArrayExpress	E-MTAB-260
BEAM	2026	32290	7	10X Genomics	
Klein	2717	24175	4	Gene expression omnibus	GSE65525
Muraro	3072	19059	11	Gene expression omnibus	GSE85241
CNIK	6647	36601	12	10X Genomics	
WB	8000	36601	13	10X Genomics	
CD19+ B Cells (CD19)	10085	32738	10	10X Genomics	
CD8+ Cytotoxic T cells (CD8)	10209	32738	10	10X Genomics

**Table 2:** Notation summary.

Notation	Meaning
*X*	Cell-gene matrix
*N*	Number of cells
*M*	Number of genes
*H*	Low-dimensional dense cell output matrix
$\widetilde{M}$	Number of random mapping nodes
$\varphi _i$	Mapping weight vector
$\zeta _i$	Mapping bias vector
$\mathcal {T}(\cdot )$	Activation function
*NO*	Noise matrix
$\widetilde{H}$	Destroyed result matrix
$\odot$	Hadamard product
*A*	Cell adjacency matrix
*MA*	Mask matrix of *A*
$A^m$	Normalization matrix of *A*
*D*	Degree matrix of *A*
$A^d$	Graph diffusion matrix of *A*
${\widetilde{H}}^{\nu _t}$	${\nu _t}$th view of $\widetilde{H}$
$AE(\cdot )$	Autoencoder network
$IGAE(\cdot )$	Improved graph autoencoder network
${{\widetilde{H}}^{\nu _t}_{AE}}$	Attribute information of cells in the $\nu _t$th view
${{\widetilde{H}}_{AE}}$	Mulit-view fusion attribute features of cells
$\widetilde{H}^{\nu _t}_{IGAE}$	Graph structure information among cells in the $\nu _t$th view
$\widetilde{H}_{IGAE}$	Mulit-view fusion graph structure features among cells
$H_I$	Initialize fusion features of cells
$H_L$	Local structure-enhanced features of $H_I$
$H_G$	Global structure-enhanced features of $H_I$
${\widetilde{H}}^*$	Fusion features of cells
${{\hat{H}}_{AE}}$	AE decoder features of ${\widetilde{H}}^*$
${{\hat{H}}_{IGAE}}$	IGAE decoder features of ${\widetilde{H}}^*$

Python package SCANPY^37^ is used to preprocess scRNA-seq data. scRNA-seq data is a single-cell gene expression matrix, where rows and columns represent cells and represent genes, respectively, with each cell having the same number of genes. In the data preprocessing step, 95% of cells with zero values of gene expression are deleted to reduce the impact of useless genes on model calculation and clustering accuracy, and the mean and variance of the normalized data range are set to 0 and 1.

### Framework of the DCRELM

The overall framework of the DCRELM is illustrated in Fig. 1. The DCRELM consists mainly of five modules: the ELM module, ELM graph distortion module, autoencoder fusion module, dual information correlation reduction module, and triplet self-supervision strategy clustering module. To better address the problem of high-dimensional sparse scRNA-seq data, first, we use the ELM to obtain low-dimensional and dense features of cells. Second, the graph distortion method is used for data augmentation, while the dynamic autoencoder fusion mechanism is employed to fuse the attribute information of cells and graph structure information among cells. Third, a dual information correlation reduction network was utilized to remove genes related to low-quality cells and genes with low expression. Last, different types of cells are effectively identified by minimizing the KL loss function of the triplet self-supervised strategy. Table 2 summarizes the notations in this paper.

### Extreme learning machine

ELM^38–40^ is known for its universal approximation capability and the hidden space created by random nonlinear feature mapping. ELM is a single hidden layer feedforward neural network (SLFN) that randomly assigns an input weight $\varphi _i$ and a hidden layer biase $\zeta _i$. The input cell-gene matrix is assumed to be $X=\left[ x_1,x_2,...,x_i,...,x_N\right] \in R^{N \times M}$, where *N* is the number of cells and *M* is the number of genes. The ELM hidden layer output matrix is expressed as follows:

1 $$\begin{aligned} H_{N\times \widetilde{M}}=\left[ \begin{array}{lll} \mathcal {T}(\varphi _1\cdot x_1+\zeta _1)&{}\cdots &{}\mathcal {T}(\varphi _{\widetilde{M}}\cdot x_1+\zeta _{\widetilde{M}})\\ \vdots &{}\cdots &{}\vdots \\ \mathcal {T}(\varphi _1\cdot x_N+\zeta _1)&{}\cdots &{}\mathcal {T}(\varphi _{\widetilde{M}}\cdot x_N+\zeta _{\widetilde{M}})\\ \end{array}\right] _{N\times \widetilde{M}} \end{aligned}$$

where $\varphi _i=\left[ \varphi _{i1,}\varphi _{i2},...,\varphi _{iM}\right] ^T$,$\ \zeta _i=\left[ \zeta _{i1},\zeta _{i2},...,\zeta _{iM}\right] ^T$, $\widetilde{M}$ is the number of random mapping nodes, and $\mathcal {T}(\cdot )$ is the activation function. In the high-dimensional and sparse feature space of scRNA-seq data, identifying cell clusters is challenging. We utilize the ELM to effectively map sparse features to low-dimensional and dense spaces, solving this problem.

### ELM graph distortion module

To further improve the generalizability and robustness of the DCRELM, we use the ELM graph distortion model to learn rich representations of cells in multiple ways. We considered feature destruction and edge perturbation, two types of distortion, in the cell graphs. Feature destruction is attribute distortion, where the noise matrix $NO\in R^{N\times \widetilde{M}}$ follows the Gaussian distribution $\mathcal {N}\left( 1,0.1\right)$. The destroyed result matrix $\widetilde{H}\in R^{N\times \widetilde{M}}$ is represented as $\widetilde{H}=H\odot NO$, where $\odot$ denotes the Hadamard product.

Moreover, there are two methods for structural distortion: edge removal based on the similarity between cells and graph diffusion. First, the paired cosine similarity of cells is calculated in the latent space. Second, the lowest 10% of linking relationships are removed, generating a mask matrix $MA\in R^{N\times N}$ based on the adjacency matrix *A* of cells. Last, *A* is normalized, i.e. $A^m=D^{-\frac{1}{2}}\left( \left( A\odot MA\right) +I\right) D^\frac{1}{2}$, where the degree matrix $D=diag(d_1,d_2,...,d_N)\in R^{N\times N}.$ In the graph diffusion step, we use the PageRank (PPR) method to transform $A^m$ into a graph diffusion matrix $A^d$. The calculation of $A^d$ is formulated as $A^d=\tau \left( I-\left( 1-\tau \right) A^m\right) ^{-1}$, where $\tau$ is the balance parameter. We employ a siamese network to obtain the feature representations of cells from two perspectives to enhance the clustering performance of the DCRELM.

### Autoencoding fusion module

As shown in Fig. 2, the autoencoder fusion module obtains the attribute information of cells and the graph structure information among cells via AE and IGAE^41^ and dynamically fuses them to obtain more suitable feature representations. An AE is a multilayer feedforward neural network with ReLU activation. The encoding and decoding of each layer are as follows:

2 $$\begin{aligned} {{\widetilde{H}}^{\nu _t}_{AE}}={AE}\left( {{\widetilde{H}}^{\nu _t}}\right) ,\ {AE}\left( {{\widetilde{H}}^{\nu _t}}\right) ^{\left( \ell \right) }=\sigma _{RELU}\left( {\mathcal {P}_1}^\ell {AE}\left( {{\widetilde{H}}^{\nu _t}}\right) ^{\left( \ell -1\right) }+{{\mathcal {B}}_1}^\ell \right) ,\ {{\hat{H}}_{AE}}^{\left( j\right) }=\sigma _{RELU}\left( {\mathcal {P}_2}^\ell {{\hat{H}}_{AE}}^{\left( j-1\right) }+{{\mathcal {B}}_2}^\ell \right) , \end{aligned}$$

where $\nu _t$ represents the $\nu _t$th view, and $\ell$ and *j* represent the $\ell$th encoder layer and *j*th decoder layer, respectively. $\mathcal {P}_1$ and ${\mathcal {B}}_1$ represent the encoding weight and biase, respectively. $\mathcal {P}_2$ and ${\mathcal {B}}_2$ represent the decoding weight and biase, respectively. $\sigma _{ReLU}$ is the ReLU activation function. ${AE}\left( {{\widetilde{H}}^{\nu _t}}\right) ^{\left( 0\right) }= {\widetilde{H}}^{\nu _t}$, ${{\hat{H}}_{AE}}^{\left( 0\right) }={\widetilde{H}}^*$, and $\hat{H}_{AE}$ is the decoding of ${\widetilde{H}}^*$. To minimize the discrepancy between ${\hat{H}}_{AE}$ and *H*, the loss function of the autoencoder (AE) is $\mathfrak {T}_{AE}=\sum {\left\| {\hat{H}}_{AE}-H \right\| }_2^2$.

IGAE is a multilayer feedforward neural network with a nonlinear activation function $\sigma$. The encoding and decoding of each layer are as follows:

3 $$\begin{aligned} \widetilde{H}^1_{IGAE}={IGAE}\left( {{\widetilde{H}}^1}\right) ,\ \widetilde{H}^2_{IGAE}={IGAE}\left( {{\widetilde{H}}^2}\right) ,\ {{\hat{H}}_{IGAE}}^{\left( {j }\right) }=\sigma \left( A^m{{\hat{H}}_{IGAE}}^{\left( {j}-1\right) }{\hat{\mathcal {W}}}^{\left( {j}\right) }\right) . \end{aligned}$$

${IGAE}\left( {{\widetilde{H}}^1}\right) ^{\left( \ell \right) }=\sigma \left( A^m{IGAE}\left( {{\widetilde{H}}^1}\right) ^{\left( \ell -1\right) }\mathcal {W}^{\left( \ell \right) }\right)$, ${IGAE}\left( {{\widetilde{H}}^2}\right) ^{\left( \ell \right) }=\sigma \left( A^d{IGAE}\left( {{\widetilde{H}}^2}\right) ^{\left( \ell -1\right) }\mathcal {W}^{\left( \ell \right) }\right)$. $\mathcal {W}^{\left( \ell \right) }$ and $\ {\hat{\mathcal {W}}}^{\left( j\right) }$ represent the learnable parameters of the $\ell$-th encoder layer and *j*-th decoder layer, respectively. $\sigma$ represents a nonlinear activation function. ${IGAE}\left( {{\widetilde{H}}^1}\right) ^{\left( 0\right) }={\widetilde{H}}^1$, ${IGAE}\left( {{\widetilde{H}}^2}\right) ^{\left( 0\right) }={\widetilde{H}}^2$ and ${{\hat{H}}_{IGAE}}^{\left( 0\right) }={\widetilde{H}}^*$. IGAE employs a mixed loss function to minimize the weighted attribute matrix and adjacency matrix, i.e. $\mathfrak {T}_{IGAE}=\mathfrak {T}_m +\gamma \mathfrak {T}_n$. $\mathfrak {T}_m=\frac{1}{2N}{\left\| {A_{norm}}H-{\hat{H}}_{IGAE} \right\| }_F^2$, $\mathfrak {T}_n=\frac{1}{2N}{\left\| {A_{norm}}-\hat{A} \right\| }_F^2$, and $A_{norm}=D^{-\frac{1}{2}}{A}D^\frac{1}{2}\in R^{N\times N}$. $\hat{H}_{IGAE}$ is the decoding of ${\widetilde{H}}^*$, $\hat{A}$ is the reconstructed adjacency matrix, and $\gamma$ is a predefined hyperparameter.

We adopt a dynamic fusion mechanism to integrate the attribute information $H_{AE}$ of each cell and the graph structure information $H_{IGAE}$ among cells, i.e. $H_I=\tau H_{AE}+\left( 1-\tau \right) H_{IGAE}$, where $H_{AE}=0.5\times \left( {\widetilde{H}}_{AE}^1+{\widetilde{H}}_{AE}^2\right)$, and $H_{IGAE}=0.5\times \left( {\widetilde{H}}_{IGAE}^1+{\widetilde{H}}_{IGAE}^2\right)$. To fully consider the local and global relationships among cells, first, we introduce the adjacency matrix *A* into $H_I$ to obtain the embedding feature $H_L$ for local structure-enhanced fusion. Second, the normalized self-correlation matrix $\mathcal {S}$ can obtain the global correlation feature $H_G$, where $H_L=A_mH_I$, $\mathcal {S}_{ij\ }=\frac{e^{\left( H_LH_L^T\right) _{ij}}}{\sum _{k=1}^{N}e^{\left( H_LH_L^T\right) _{ik}}}$, and $H_G=\mathcal {S}H_L$. Last, we use a local structure to enhance features and global correlation features to extract latent features, i.e. ${\widetilde{H}}^*=\beta {H_G}+H_L$, where $\beta$ is a learnable parameter.

### Dual information correlation reduction

We use a dual information correlation reduction network (DICR) to remove redundant information and improve the discriminative ability of the learned embedded features. Specifically, dual information correlation reduction is reduced in two ways: sample-level correlation reduction and feature-level correlation reduction.

First, we calculate the cross-view sample correlation matrix $\mathcal {S}^\mathcal {C}$. $\mathcal {S}_{ij}^\mathcal {C}=\frac{\left( H_i^{\nu _1}\right) \left( H_j^{\nu _2}\right) ^T}{\left\| H_i^{\nu _1} \right\| \left\| H_j^{\nu _2} \right\| },\forall i,j\in \left[ 1,N\right]$, where $H^{\nu _1}$ and $H^{\nu _2}$ are two view nodes embedded through the siamese network. The cross-view correlation matrix $\mathfrak {T}_\mathcal {C}$ is normalized, i.e. $\mathfrak {T}_\mathcal {C}=\frac{1}{N^2}\sum \left( \mathcal {S}^\mathcal {C}-I\right) ^2 \nonumber$. The purpose was to pull in two samples of the same dimension and pull out samples of different dimensions.

Second, the correlation reduction of feature levels is divided into three steps. (1) The readout function $\mathcal {R}\left( \cdot \right)$ is used to embed $H^{\nu _1}$ and $H^{\nu _2}$ from $R^{N \times d}$ mapped to $R^{k \times d}$: ${\widetilde{H}}^{\nu _t}=\mathcal {R}\left( H^{\nu _t}\right) ,t=1,2.$ (2) The cosine similarity is calculated based on ${\widetilde{H}}_i^{\nu _1}$ and ${\widetilde{H}}_j^{\nu _2}$: $\mathcal {S}_{ij}^\mathfrak {F}=\frac{\left( {\widetilde{H}}_{:j}^{\nu _1}\right) ^T\left( {\widetilde{H}}_{:i}^{\nu _2}\right) }{\left\| {\widetilde{H}}_i^{\nu _1} \right\| \left\| {\widetilde{H}}_j^{\nu _2} \right\| },\forall i,j\in \left[ 1,...,d\right]$, where ${\widetilde{H}}_{:j}^{\nu _1}$ represents the *j*-th column of ${\widetilde{H}}^{\nu _1}$ and ${\widetilde{H}}_{:i}^{\nu _2}$ represents the *i*-th column of ${\widetilde{H}}^{\nu _2}$. (3) We perform normalization processing to pull in two features of the same dimension and pull out features of different dimensions, i.e. $\mathfrak {T}_\mathfrak {F}=\frac{1}{d^2}\sum \left( \mathcal {S}^\mathfrak {F}-\widetilde{I}\right) ^2.$ We obtain the latent features $H=\frac{1}{2}\left( H^{\nu _1}+H^{\nu _2}\right)$. Therefore, considering information reduction from two dimensions can further remove redundant information.

### Clustering module

The DCRELM employs a triplet self-supervised strategy to enhance clustering performance, which simultaneously leverages the target distribution to enhance the guidance for the AE and IGAE.

We utilize the *t*-distribution to compute the similarity between the samples and the clustering centres in the fusion embedding $\widetilde{H}$. This similarity measurement helps capture the relationship between the samples and the clustering centres during the clustering process. Fusion embedding $\widetilde{H}$ integrates AE and IGAE information to generate a target distribution. The calculation process is described as follows: $q_{ij}=\frac{{\left( 1+{\left\| {{\widetilde{H}}^*}_i-\mu _j \right\| }^2/\upsilon \right) }^{-\frac{\upsilon +1}{2}}}{{\sum }_{j^\prime }{\left( 1+{\left\| {{\widetilde{H}}^*}_i-\mu _{j^\prime } \right\| }^2 / \upsilon \right) }^{-\frac{\upsilon +1}{2}}}$, where the degree of freedom for the Student’s *t*-distribution is denoted by $\upsilon$, while $q_{ij}$ represents the probability of assigning the *i*-th node to the *j*-th centre. This probability, which is referred to as a soft assignment, quantifies the likelihood of the *i*-th node belonging to the *j*-th centre. We normalize the frequency of each cluster based on $q_{ij}$ and obtain the calculation method for $p_{ij}$ as follows: $p_{ij}=\frac{{q^2}_{ij}/\sum _{i} q_{ij}}{\sum _{j^\prime }\left( {q^2}_{ij^\prime }/\sum _{i} q_{ij^\prime }\right) }$. The distribution $q^\prime$ of $H_{AE}$ and the distribution $q^{\prime \prime }$ of $H_{IGAE}$ are calculated in the same way as the distribution of ${\widetilde{H}}^*$ is calculated. We adopt the KL-divergence and designate the triplet self-supervised strategy clustering loss as:

4 $$\begin{aligned} \mathfrak {T}_{KL}=\sum _{i}\sum _{j}{p_{ij}log\frac{p_{ij}}{\left( q_{ij}+{q^\prime }_{ij}+{q^{\prime \prime }}_{ij}\right) /3}} \end{aligned}$$

### Objective function

As shown in Eq. (5), the learning objective function of the DCRELM comprises three main components: the reconstruction loss of AE and IGAE, the DICR module, and the clustering model. These components collectively contribute to the learning process of the DCRELM. The DICR module includes $\mathfrak {T}_\mathcal {C}$ loss, $\mathfrak {T}_\mathfrak {F}$ loss, and $\mathfrak {T}_\mathcal {R}$ loss, where $\mathfrak {T}_\mathcal {R}=JSD(H,\widetilde{A}H)$, and it is aimed at alleviating oversmoothing. $JSD (\cdot )$ refers to the Jensen–Shannon divergence. $\mathfrak {T}_{KL}$ is the clustering loss function. $\varepsilon$ and $\lambda$ are hyperparameters.

5 $$\begin{aligned} \mathfrak {T}={\underbrace{{\mathfrak {T}_{AE}+\mathfrak {T}_{IGAE}}}_{Reconstruction}}+{\underbrace{{\mathfrak {T}_\mathcal {C}+\mathfrak {T}_\mathfrak {F}+\varepsilon \mathfrak {T}_\mathcal {R}}}_{DICR}}+{\underbrace{{{\lambda \mathfrak {T}}_{KL}}}_{Clustering}} \end{aligned}$$

### Time complexity analysis

DCRELM consists of five parts: ELM module, ELM graph distortion module, dual information correlation reduction module, autoencoder module, and autoencoder fusion module. These five parts correspond to time complexities $$O(N*M*\widetilde{M})$$, $$O(N^2)$$, $$O(N^2*d)$$, $$O(N*M*d)$$, and $$O(N^2*d)$$, where *N* is the number of cells, *M* is the number of genes, $$\widetilde{M}$$ is the number of random mapping nodes, and *d* is the embedding size. Therefore, the total time complexity of DCRELM is $$O(N*M*\widetilde{M})+O(N^2*d)+O(N*M*d)$$, where $$\widetilde{M}$$ and *d* are much smaller than *M*. Overall, DCRELM significantly reduces the dimensionality of gene representation and can better handle larger scale scRNA-seq datasets.

### Implementation and parameter settings

This paper conducts experiments using PyTorch to execute the DCRELM in a Python 3.8 environment. The number of randomly mapped nodes is selected from $\{200, 500, 1000, 1500, 2000\}$. The number of nodes in the first three layers of the AE encoding layer is selected from $\left\{ 256, 512, 1024, 2048\right\}$, and the number of nodes in the last layer is equal to the number of randomly mapped nodes. The number of nodes in the first two layers of the IGAE encoding layer is selected from $\left\{ 256, 512, 1024, 2048\right\}$, and the number of nodes in the last layer is equal to the number of randomly mapped nodes. The DCRELM is trained using Adam with 2000 epochs and a learning rate of 0.0001. All the experiments were conducted on an NVIDIA A40 (48G).

### Evaluation metrics

We use three evaluation metrics—the normalized mutual information (NMI), adjusted rand index (ARI), and $F_1$—to measure the clustering performance of the clustering methods. The NMI is utilized to measure the similarity of the clustering results and combines the concepts of information entropy and mutual information. The ARI is employed to quantify the agreement between the predicted clusters and the true clusters. $F_1$ measures the classification performance of the algorithms.

## Results and discussion

### Comparison of algorithm clustering performance

In this section, we conduct clustering experiments on 12 real scRNA-seq datasets and compare them with six state-of-the-art, single-cell clustering methods, namely, scDeepCluster^20^, GraphSCC^34^, scGNN^32^, DREAM^31^, scDCCA^29^, and scDFC^35^. Furthermore, we employ three evaluation metrics, namely, the NMI, ARI, and $F_1$, to assess the performance of each method.

Tables 3, 4 and 5 show the experimental results of seven methods on 12 real scRNA-seq datasets. The best results are highlighted in bold. As shown in Tables 3, 4 and 5, the DCRELM achieves the best clustering performance in most datasets. With the exception of three datasets, the DCRELM has the highest NMI and ranks second in terms of the ARI among all the algorithms. Although the DCRELM is not the highest on the Kolo, WB, or CNIK datasets, it still performs in the top three. In terms of $F_1$, the DCRELM significantly outperforms all the other algorithms. On the Klein and Muraro datasets, the DCRELM exhibits significant improvements in terms of the NMI and ARI compared to the scGNN. Overall, the DCRELM outperforms the other methods.

**Table 3:** NMI of the DCRELM and six comparison methods on 12 datasets.

Datasets	scDeepCluster	GraphSCC	scGNN	DREAM	scDCCA	scDFC	DCRELM
Human	0.7766	0.3502	0.7500	0.8421	0.5786	0.7230	**0.8575**
Yeo	0.6867	0.2214	0.6453	0.5184	0.6940	0.6265	**0.6996**
Ning	0.1962	0.0085	0.2335	0.0669	0.0617	0.0819	**0.2856**
Lawor	0.5404	0.5859	0.5758	0.4703	0.3840	0.6943	**0.7137**
Kolo	0.0575	0.0362	0.1037	**0.4350**	0.0561	0.0587	0.2497
BEAM	0.2415	0.4396	0.3570	0.4510	0.0940	0.2661	**0.4772**
Klein	0.7409	0.6297	0.4539	0.5728	0.7680	0.5996	**0.7785**
Muraro	0.7254	0.5927	0.5277	0.5435	0.3902	0.4782	**0.7583**
WB	0.3711	0.4592	0.3621	0.5042	0.4839	**0.5356**	0.4740
CNIK	0.2938	0.4234	0.3513	**0.5790**	0.4305	0.4543	0.4330
CD19	0.1019	0.0928	0.1363	0.1942	0.1921	–	**0.2119**
CD8	0.0808	0.0795	0.1416	0.1657	0.1768	–	**0.3220**

The optimal values are shown in bold.

**Table 4:** ARI of the DCRELM and six comparison methods on 12 datasets.

Datasets	scDeepCluster	GraphSCC	scGNN	DREAM	scDCCA	scDFC	DCRELM
Human	0.6525	0.1665	0.1973	0.7682	0.4071	0.6271	**0.8166**
Yeo	0.6249	0.2219	0.5661	0.4541	0.6793	0.6542	**0.7346**
Ning	0.0784	0.0067	0.1406	0.0357	0.0256	0.0204	**0.2608**
Lawor	0.4105	0.4092	0.4133	0.3148	0.3309	0.7617	**0.8287**
Kolo	0.0622	0.0000	0.0652	**0.3902**	0.0504	0.0511	0.2793
BEAM	0.1916	0.3348	0.1599	0.3543	0.2139	0.0188	**0.4390**
Klein	0.7205	0.5156	0.3124	0.5014	0.7680	0.4095	**0.8053**
Muraro	0.6584	0.5105	0.4586	0.4531	0.1682	0.2439	**0.7267**
WB	0.1722	0.1848	0.0961	0.2415	0.2511	0.2548	**0.2993**
CNIK	0.2143	0.1973	0.1426	**0.3782**	0.2419	0.1757	0.2654
CD19	0.0499	0.0424	0.0217	0.0875	0.1227	–	**0.1490**
CD8	0.0512	0.0328	0.0257	0.0979	0.0883	–	**0.2155**

The optimal values are shown in bold.

**Table 5:** $F_1$ of the DCRELM and six comparison methods on 12 datasets.

Datasets	scDeepCluster	GraphSCC	scGNN	DREAM	scDCCA	scDFC	DCRELM
Human	0.0187	0.0251	0.0476	0.0000	0.0651	0.1672	**0.8851**
Yeo	0.0123	0.1810	0.1584	0.2731	0.1961	0.2041	**0.8715**
Ning	0.1286	0.1582	0.0320	0.1997	0.1643	0.1601	**0.4272**
Lawor	0.2439	0.0407	0.0526	0.0096	0.0869	0.0266	**0.4835**
Kolo	0.2434	0.1969	0.0236	0.0905	0.2873	0.3532	**0.5991**
BEAM	0.1428	0.1980	0.0287	0.1385	0.0940	0.0295	**0.6116**
Klein	0.0078	0.3039	0.1895	0.0488	0.1839	0.0015	**0.9041**
Muraro	0.0113	0.1379	0.0177	0.0491	0.0729	0.0643	**0.6819**
WB	0.0499	0.0767	0.0035	0.0897	0.0441	0.0084	**0.6819**
CNIK	0.0248	0.1058	0.0110	0.2157	0.0712	0.0557	**0.5017**
CD19	0.0383	0.0593	0.0682	0.1205	0.0682	–	**0.3136**
CD8	0.0718	0.1107	0.0032	0.1088	0.0800	–	**0.3018**

The optimal values are shown in bold.

**Figure 3:**
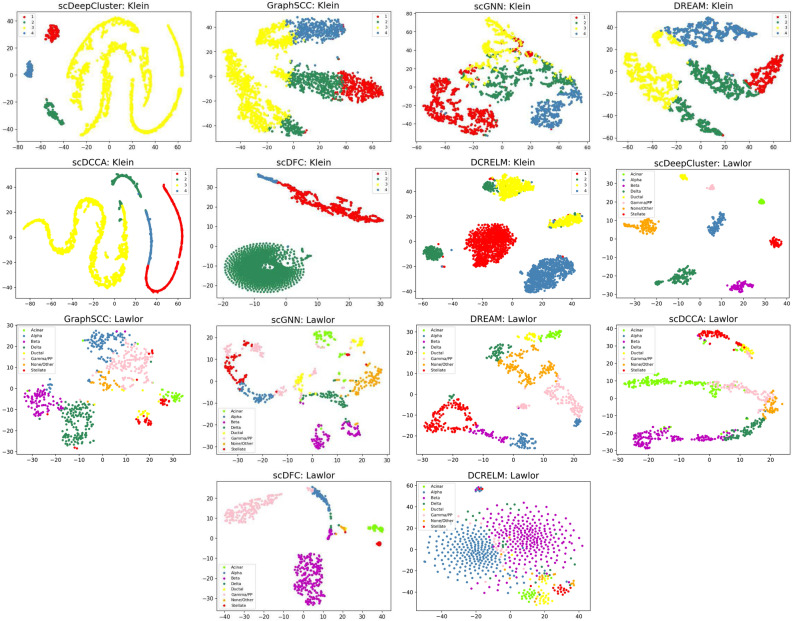
Visualization of the prediction results of seven clustering methods on the Klein and Lawlor datasets.

To visualize the clustering results of the seven clustering methods, we choose a smaller scale real dataset Lawlor and a larger scale real dataset Klein, and use t-SNE^42^ to project the clustering results of each clustering method into two-dimensional space. We compared the DCRELM with the other six clustering methods on the Lawlor and Klein datasets. As shown in Fig. 3, the different cell subtypes predicted by the DCRELM exhibit distinct boundaries, enabling a distinction among different cell subtypes with only a few remaining samples and mixtures. In contrast, many cell clusters identified by the other seven methods have yet to be identified and include a greater mixture of different cell subtypes. The analysis indicates that the DCRELM can effectively reduce the distance among cells within clusters of the same class.

### Model stability

**Figure 4:**
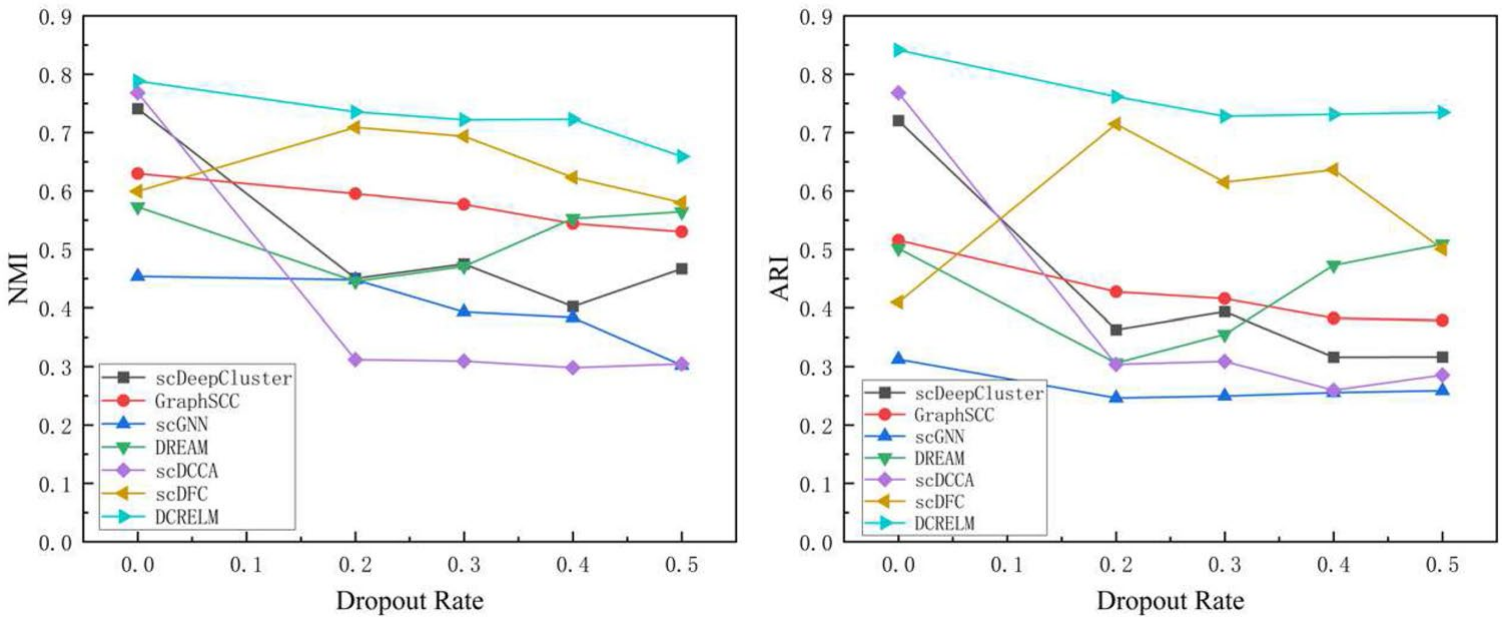
Change in the NMI and ARI for each method on the Klein datasets with 20%, 30%, 40%, and 50% dropout rates.

To further verify the stability and robustness of the DCRELM, we conduct dropout experimental analysis on the dataset Klein and randomly select 20%, 30%, 40%, and 50% of the nonzero values to set zero in *X*. We use two evaluation metrics, namely, the NMI and ARI, to measure the clustering performance of the DCRELM and six comparison methods. The experimental results are shown in Fig. 4, which reveals that all the algorithms are affected by noise interference. Moreover, scDeepCluster and scDCCA are more affected by noise, resulting in significant degradation of their clustering performance. GraphSCC is generally relatively stable, but its performance is not optimal. The DCRELM has less interference from noise, demonstrating strong stability and robustness.

### Parameter analysis

**Figure 5:**
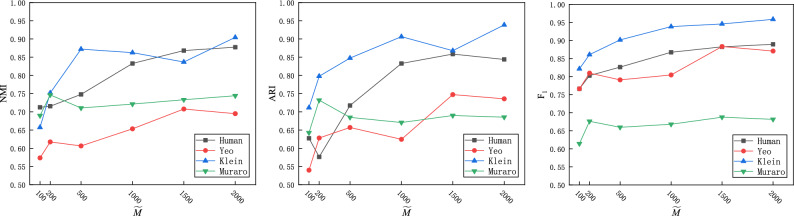
Impact of latent feature $\widetilde{M}$ dimension on the clustering performance of the DCRELM.

To obtain low-dimensional and dense cell gene expression features, we use the parameter $\widetilde{M}$ to control the number of hidden layer nodes. The parameter selection range of $\widetilde{M}$ is $\{100, 200, 500, 1000, 1500, 2000\}$. Figure 5 shows the effect of $\widetilde{M}$ in terms of the NMI, ARI, and $F_1$ of the DCRELM on four datasets: Human, Yeo, Klein, and Muraro. Figure 5 shows that the clustering performance of the DCRELM varies with respect to $\widetilde{M}$ on the four datasets. For example, the DCRELM is not very sensitive to $\widetilde{M}$ on the Muraro dataset, while it is sensitive to $\widetilde{M}$ on the Human dataset. Therefore, the selection of the appropriate $\widetilde{M}$ value plays an important role in the clustering performance of the DCRELM.

**Figure 6:**
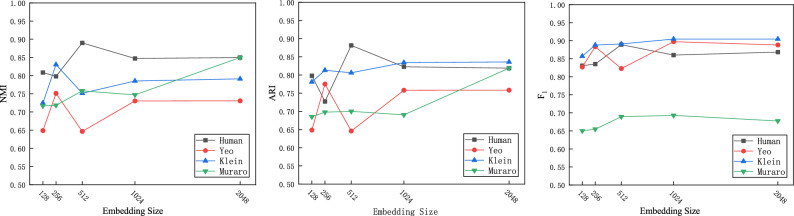
Impact of the embedding size on the clustering performance of the DCRELM across four datasets.

To obtain the effective attributes and graph structure information of cells, we use embedding dimensions to control the number of nodes in the network layer for the AE and IGAE. The selection range for embedding dimensions is $\{128, 256, 512, 1024,2048\}$. Figure 6 shows the impact of the parameter embedding dimension on the clustering results of the DCRELM on the four datasets. Based on Fig. 6, for datasets with sample sizes smaller than 1000, the optimal embedding dimension size for the AE and IGAE network layers is set to 256. For datasets with sample sizes larger than 1000, the optimal embedding dimension size for the AE and IGAE network layers is set to 2048. Therefore, the appropriate embedding dimension is related to the sample number of datasets.

### Ablation experiments

We conduct ablation experiments on four datasets: Human, Yeo, Klein, and Muraro. Dual information correlation reduction, dynamic autoencoder fusion, and graph distortion modules play important roles in improving the clustering performance of the DCRELM. To analyse the impact of each module on the clustering performance of the DCRELM, four variants of the DCRELM are constructed. DCRELM-CR refers to the variant of the DCRELM in which the dual information correlation module is removed. DCRELM-DF refers to the variant of the DCRELM in which IGAE is removed while retaining the AE. DCRELM-N refers to the variant of the DCRELM in which feature destruction from the graph distortion module is removed. DCRELM-E refers to the variant of the DCRELM where edge disturbances from the graph distortion module are removed.

**Figure 7:**
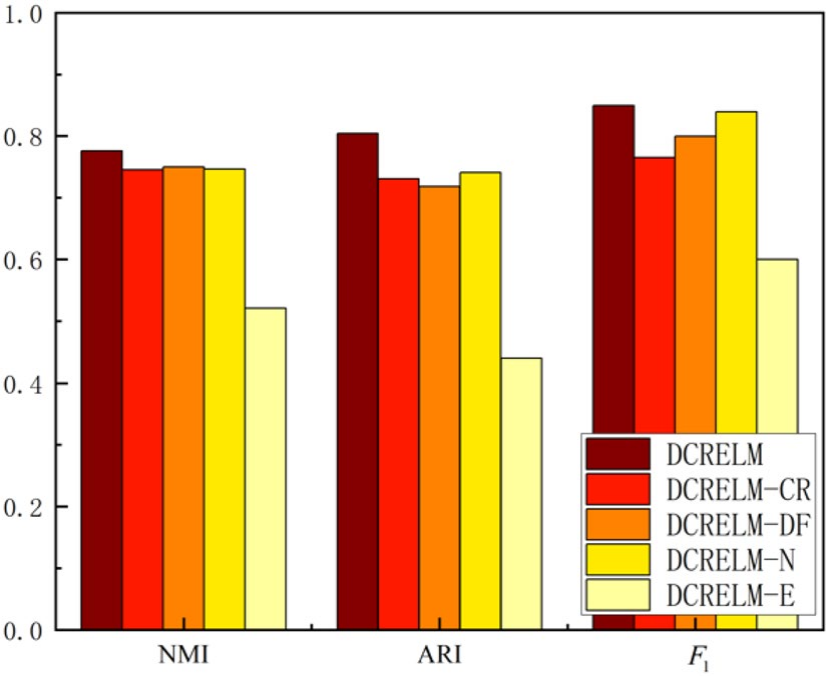
Comparative analysis of the clustering performance between the DCRELM and its four variants.

As shown in Fig. 7, due to the removal of the dual information correlation reduction module, DCRELM-CR could not effectively remove low-quality cells or genes with low expression. Therefore, the NMI, ARI, and $F_1$ of DCRELM-CR are lower than those of the DCRELM. Due to the removal of the dynamic autoencoder fusion, DCRELM-DF cannot effectively utilize the graph structure information of the fused cells. Therefore, the NMI, ARI, and $F_1$ of DCRELM-DF are lower than those of the DCRELM. Due to the removal of feature destruction and edge disturbance in the graph distortion module, DCRELM-N and DCRELM-E exhibit lower robustness than the DCRELM.

## Conclusion

In this paper, we propose a new deep clustering method, the DCRELM, for scRNA-seq data. This method obtains low-dimensional and dense gene representations through an ELM random mapping space and then uses a graph distortion module to improve the robustness and uncertainty of the model. The dynamic fusion of dense-cell gene representations with cell attribute information and graph structure information helps establish connections among cells and among genes. We employ dual information correlation reduction to filter out redundant information and noise at both the cellular level and gene level. Additionally, we utilize a triple, self-supervised learning mechanism to further enhance the clustering performance. Extensive experiments demonstrate that the DCRELM outperforms the other comparison methods. In the future, we will consider multimodal data clustering, integrating data from different levels to more comprehensively describe the heterogeneity of single cells.

## Data Availability

These six scRNA-seq datasets analysed during the current study are available in the Gene Expression Omnibus (GEO) repository with accession numbers of GSE36552 (Human), GSE85908 (Yeo), GSE64016 (Ning), GSE86473 (Lawlor), GSE65525 (Klein), and GSE85241 (Mauro). The Kolo dataset analysed during the current study are available in the ArrayExpress repository with an accession number of E-MTAB-260. The BEAM, CNIK, WB, CD19, and CD8 analysed during the current study are available in the 10X Genomics website repository, https://www.10xgenomics.com/datasets/2k-transgenic-hel-mouse-splenocytes-beam-ab-2-standard (BEAM), https://www.10xgenomics.com/datasets/5k-human-pancreatic-tumor-isolated-with-chromium-nuclei-isolation-kit-3-1-standard (CNIK), https://www.10xgenomics.com/datasets/whole-blood-rbc-lysis-for-pbmcs-and-neutrophils-granulocytes-5-3-1-standard (WB), https://www.10xgenomics.com/datasets/cd-19-plus-b-cells-1-standard-1-1-0 (CD19), and https://www.10xgenomics.com/datasets/cd-8-plus-cytotoxic-t-cells-1-standard-1-1-0 (CD8).
